# Protein-Mediated Biotemplating on the Nanoscale

**DOI:** 10.3390/biomimetics2030014

**Published:** 2017-08-08

**Authors:** Amihay Freeman

**Affiliations:** Department of Molecular Microbiology and Biotechnology, Faculty of Life Sciences, Tel Aviv University, Tel Aviv 69978, Israel; amihayf@post.tau.ac.il

**Keywords:** protein, biotemplate, nanoscale, array, nanoparticle

## Abstract

Purified proteins offer a homogeneous population of biological nanoparticles, equipped in many cases with specific binding sites enabling the directed self-assembly of envisaged one-, two- or three-dimensional arrays. These arrays may serve as nanoscale biotemplates for the preparation of novel functional composite materials, which exhibit potential applications, especially in the fields of nanoelectronics and optical devices. This review provides an overview of the field of protein-mediated biotemplating, focussing on achievements made throughout the past decade. It is comprised of seven sections designed according to the size and configuration of the protein-made biotemplate. Each section describes the design and size of the biotemplate, the resulting hybrid structures, the fabrication methodology, the analytical tools employed for the structural analysis of the hybrids obtained, and, finally, their claimed/intended applications and a feasibility demonstration (whenever available). In conclusion, a short assessment of the overall status of the achievements already made vs. the future challenges of this field is provided.

## 1. Introduction

Purified proteins offer a homogeneous population of biological nanoparticles, equipped in many cases with specific binding sites enabling directed self-assembly into one-, two- or three-dimensional arrays. These arrays may serve as biotemplates for the preparation of novel functional composite materials on the nanoscale, using either specific or site-directed chemical binding, with the potential for expansion by genetic or chemical manipulations.

The potential inherent in protein-mediated biotemplating has attracted much attention throughout the past two decades (for a review, see [1]). Several other reviews dedicated to specific categories of nanocomposites obtained by protein-mediated biotemplating were later published throughout the past decade, including electronic and optical materials [2], inorganic nanoparticles [3], or metal and metal oxide nanoparticles [4]. For an appropriate comprehension of the extent of this field, it should be noted that biomimetic applications of protein-mediated biotemplating represent only a fraction of the potential applications inherent to bioinspired templating systems (e.g., those inspired by natural inorganic structures or biotemplating by polysaccharides [5,6]).

This review provides an overview of the field of protein-mediated nanoscale biotemplating, focused on the achievements made throughout the past decade. The review is organized into sections according to the size and configuration of the protein-made biotemplate ranging from single protein soluble molecules and supramolecular protein “cages” to two-dimensional (2D) protein arrays, protein fibers and tubes, viral envelopes, three-dimensional (3D) protein crystals, and fragments of microbial cells.

Each section describes the structure and size of the biotemplates employed, the fabrication methodology, the analytical tools used for its structural analysis, its claimed/intended applications and a feasibility demonstration (whenever available).

## 2. Single Soluble Protein Molecules Biotemplating

**Figure 1:**
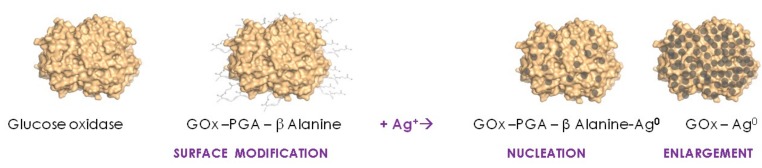
Electroless deposition and nucleation/enlargement of metallic silver on the surface of the enzyme glucose oxidase (GOx). PGA: Polyglutaraldehyde.

Dagan-Moscovich et al. [7] initiated a series of studies on single soluble protein molecules as biotemplates for the fabrication and application of protein–metal hybrids comprised of biologically active protein molecule cores coated with a porous, thin metallic layer. The methodology developed was based first on the nucleation/enlargement mechanism of the electroless deposition of silver, directed to the surface of a protein by pre-decoration with a silver-reducing polymer (short polymeric glutaraldehyde linear chains displaying Schiff bases with β-alanine groups) and exposure to silver ions. Following this nucleation step, the atomic silver nuclei thus formed were gradually enlarged by the controlled addition of a solution containing an additional amount of the reducing polymer and silver ions (non-bound reagents were removed by ultrafiltration following each step), resulting in the process schematically described in Figure 1.

**Figure 2:**
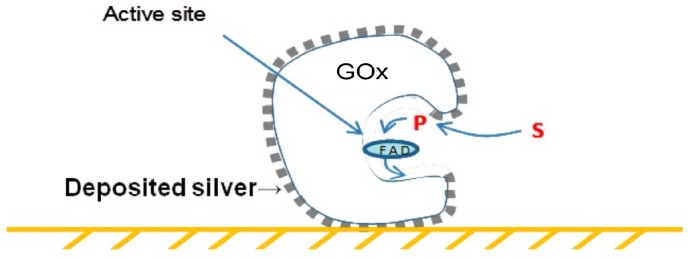
Schematic side view of the glucose oxidase (GOx)–silver hybrid “wired” to the surface of a gold electrode. FAD: Flavin adenine dinucleotide (oxidized/quinone form); P: Product; S: Substrate.

The construction of a conducting metallic silver coating on the surface of the oxidoreductase enzyme glucose oxidase (GOx), a 5 × 8 nm ‘peanut-like’ dimer, enabled the nanowiring of its active site to platinum electrodes, resulting in the oxygen-independent direct determination of glucose by electrochemical biosensors [7], as schematically described in Figure 2. The structural and functional analyses were performed using high-resolution transmission electron microscopy (HRTEM) and electrochemical biosensing.

The biotemplating approach for electroless metal deposition directed to the surface of single soluble protein molecules was readily expanded from silver deposition to the deposition of other metals [8]. Glucose oxidase molecules were first modified with an excess of short polyglutaraldehyde (PGA) polymeric chains. Following the removal of the unbound polymer, the wide-spectrum metal ion complexing agent (iminodiacetate) was chemically attached to the PGA chains displayed on the surface to introduce the subsequently metallic nucleation sites. The nucleation/enlargement of the electroless deposition of metals was subsequently carried out by using hypophosphite as a soluble, wide-spectrum, metal ion reducing agent and by the removal of the non-bound excess of reagents by ultrafiltration following each step. The construction of a porous conducting palladium layer was thus readily demonstrated, enabling the nanowiring of the GOx binding site to platinum electrodes. The structural and functional analyses were performed using HRTEM and electrochemical biosensing.

**Figure 3:**
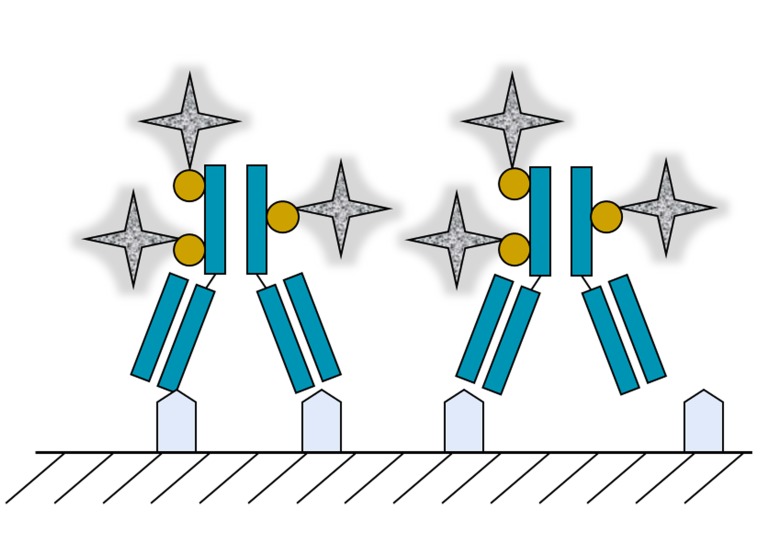
Schematic description of silver–avidin (gray) hybrids bound to biotinylated (yellow) antibodies (Erbitux®/cetuximab; blue), labeling epidermal growth factor receptors (EGFR) overexpressed on the surface of cancer cells.

By changing the biologically active protein core from GOx to the binding protein avidin, which is capable of specifically binding the vitamin biotin, additional applications of metallic silver-coated soluble protein molecules became available: imaging by HRTEM without staining. Mor et al. [9] demonstrated the feasibility of using a silver–avidin hybrid for the molecular imaging of markers overexpressed on the surface of cancer cells, pre-labeled with biotinylated monoclonal antibodies, as illustrated in Figure 3.

The range of applications of silver–enzyme hybrids was expanded further to antimicrobial applications aiming at the prevention of microbial biofilm formation, by affecting the on-site enzymatically attenuated release of silver ions, generating in situ antimicrobial activity. ‘Zone of inhibition’ antimicrobial studies demonstrated the feasibility of using these hybrids as wide-spectrum antimicrobial agents, especially as potential antifungal agents against skin and hair fungal contaminations [10].

Veelders and Essen [11] demonstrated ‘on-site’ biotemplating on single protein molecules by the formation of complex polynuclear gadolinium clusters on an acidic site displayed on the surface of fungal cell adhesion domain of Flo5A protein (a C-type lectin). This site mediated the formation of ‘site-templated’ heptanuclear, or larger, gadolinium-oxo clusters in a soaking-time dependent manner. These findings appear to pave the way for the future preparation of improved biocompatible magnetic resonance imaging (MRI) contrasting agents. The structural analysis information was collected from X-ray diffraction data obtained from protein complex crystals.

Kong et al. [12] described protein-mediated biotemplating based on protein molecules acting as a capping agent for the preparation of CdTe quantum dots (QDs) by using the enzyme ribonuclease A. The conjugation of RGD (arginine–glycine–aspartic acid) to the protein’s surface yielded a series of QDs associated with RGD-decorated ribonucleases (RNases), emitting different colors and applicable as a diagnostic tool. The labeling of gastric cancer cells was successfully demonstrated using confocal microscopy. In a subsequent study, Chen et al. [13] demonstrated the controlled preparation of Ag_2_S nanoparticles (NPs) ~20 nm in diameter, enabled by RNase A capping, as hollow or full spheres with a claimed potential application as a drug delivery vehicle. The structural characterization of the QDs and Ag_2_S NPs thus obtained was carried out by high-resolution electron microscopy and spectral analysis.

Three recent reports have described biomineralization processes directed by protein molecules and designed for biomedical applications, including (i) the formation of albumin-mediated platinum nanocrystals for tomography imaging [14]; (ii) silk fibroin-based template mediating silica-coated protein assembly for anticancer drug carriers [15]; and (iii) recombinant collagen-directed biotemplating of hematite nanocrystals [16].

## 3. Protein “Cages” as Biotemplates

Ferritin—a natural, spherical, protein-made nanoscale “cage”—serves as an iron storage protein in bacteria, plants, animals and humans. In eukaryotic cells, ferritin is comprised of 24 subunits forming a hollow spherical shell with an inner diameter of 8 nm [17,18]). The hollow spherical ferritin shell is decorated on the inside with negatively charged acidic amino acid residues, triggering the internal accumulation of iron ions or other related biomineralization processes.

The currently available biochemical data on the molecular mechanisms involved in these processes has inspired an exploration of the potential biomedical applications of a series of ferritin and mutated ferritin as protein cages for the preparation of hybrids by biotemplating. The templating of metals, alloys and metal ion salts, including iron, silver, gold, palladium, cadmium, cobalt, platinum and copper, were described (for comprehensive reviews summing up ferritin biochemical characteristics, its mediated biotemplating and their biomedical applications, see [17,18]). It is pertinent to note that the use of the ferritin cage for the encapsulation of bioactive nutrients has also recently been reviewed [19]; see also [20]. In addition to the use of ferritin in solution, potential immobilized ferritin-mediated biotemplated hybrids have also been suggested, by the construction of 2D arrays of ferritin molecules on electrodes [21] as well as the directed construction of a 3D array enabled by ferritin ornamentation with Ti-binding peptide [22].

The demonstrated feasibility of using the cavity, 7 nm in diameter, of natural ferritin for biotemplating has triggered efforts to develop analogous, smaller protein cages made of other proteins, resulting in CdS and CdSe NPs biotemplated inside the 4.5 nm diameter cavity of a cage-shaped protein derived from *Listeria innocua* [23,24], as well as their self-assembly into 2D arrays (characterized by HRTEM).

The use of the cage-forming protein clathrin as a biotemplate for the synthesis of metallic NPs was also described, enabling the preparation of either homogeneous or bimetallic silver–gold or silver–copper NPs, albeit in sizes larger than those available by ferritin-mediated biotemplating (characterized by HRTEM) [25].

Voet et al. [26] demonstrated the feasibility of biotemplating the nanocrystal formation of 7·CdCl_2_ molecules by using a small de novo designed protein, self-assembled as a “nanoreactor” for biomineralization. Behrens et al. [27] described the use of an aspen plant protein which was self-assembled into a ring-shaped template, providing a cavity of 2–3 nm (internal diameter), enabling the biotemplating of palladium NPs of ~3 nm in diameter (characterized by HRTEM).

Zhou et al. [28] described the growth mechanism and kinetics of semiconducting CdS nanocrystals of 2.5 to 5.8 nm in diameter grown inside a genetically engineered virus-like particle (characterized by transmission electron microscopy (TEM) and spectrophotometric analysis). Gold sub-nanocluster nucleation within a crystalline protein cage was recently described by Maity et al. [29]. Directing gold nucleation to the outer surface of a protein cage, made of the bacteriophage P22 protein, was also recently demonstrated [30].

## 4. Two-Dimensional Protein Arrays as Biotemplates

The pioneering work of Slytr et al. [31] on the in vitro reconstitution of self-assembled 2D bacterial S-layers paved the way for a series of studies on 2D protein array-mediated biotemplating. Throughout the period covered in this review, a series of studies were carried out on 2D bacterial surface (S)-layer biotemplating, including a preliminary study by Mark et al. [32] describing the bionanofabrication of silicon nanopillar structures using gold NPs pre-templated onto a 2D bacterial S-layer. In a subsequent study from this group, germanium nanowires were also grown from pre-synthesized gold NPs biotemplated on the surface of a bacterial S-layer, verified by scanning electron microscopy (SEM) structural analysis [33].

The elucidation of the mechanism underlying the biotemplating of FePt NPs from the gas phase into a 2D array on the surface of a bacterial S-layer was presented by Queitsch et al. [34] and verified by TEM structural analysis. Varga et al. [35] reported the capability of the recombinant S-layer to display streptavidin 2D arrays in a defined order and orientation, exhibiting the biotemplating of biotinylated QDs for biosensing applications (verified by TEM).

Valero et al. [36] demonstrated the biotemplating of CrNi NPs as 2D layers, reflecting the same pattern of its parent bacterial S-layer template, as verified by HRTEM, energy-dispersive X-ray spectroscopy (EDXS) and infrared radiation (IR) structural analysis, with a potential application for molecular spintronics.

Two-dimensional protein arrays other than bacterial S-layers were also used as biotemplates: Shindel and coworkers [37,38,39] explored the use of 2D streptavidin arrays as biotemplates for the directed 2D positioning of biotinylated, pre-prepared gold NPs. Gold nanoparticle arrays thus obtained were characterized by atomic force microscopy (AFM), spectrophotometry and fluorescence analysis.

Bovine serum albumin adsorbed as a 2D monolayer on the surface of TiO_2_ nanotubes enabled directed gold deposition, resulting in a hybrid exhibiting enhanced electron transfer between heme proteins and electrodes [40].

Galloway et al. [41] and Bird et al. [42] explored the use of a 2D self-assembled monolayer (SAM) array of the magnetosome membrane specific 6 (Mms6) protein derived from the magnetotactic bacterium *Magnetospirillum magneticum* for biotemplating of magnetic nanoparticle arrays; characterized by TEM, SEM and AFM, with potential applications in the manufacture of electric circuits.

## 5. Protein-Made Fibers and Tubes as Biotemplates

The data available in the literature on self-assembly of proteins into fibers and tubes has paved the way for the exploitation of the inherent potential of using a series of readily available self-assembled protein fibers as biotemplates.

Colby et al. [43] described the use of α-synuclein fibrils as a template for coatings made of palladium, gold or copper nanoparticle chains. The resulting nanoparticle chains exhibited diameter sizes within the range of 50–200 nm (as verified by TEM, EDXS and electron energy loss spectroscopy (EELS) structural analysis), leading to potential applications in nanophotonic devices, storage media or quantum dot assays.

Malisauskas et al. [44] described the use of amyloid fibers obtained by the self-assembly of lysozyme into tubular templates for the synthesis of silver wires—1 nm in diameter—enabled by the presence of 2,2,2-trifluroethanol affecting the simultaneous fibril assembly of the protein template and in situ ionic silver reduction within the biotemplate’s hollow channels (characterized by AFM, spectrophotometry and fluorescence measurements) into nanowires obtained following the enzymatic proteolytic digestion of the protein-made template.

Slocik et al. [45] described the use of filaments made of the γ-pre-folding protein derived from the hyperthermophile *Methanocaldococcus jannaschii* as a biotemplate decorated with silver, gold, palladium or platinum NPs, by electroless deposition using NaBH_4_ as a reducer of pre-adsorbed metal ions. The coated protein filaments thus obtained were characterized by TEM, spectral and electrical analysis, with the intended application of metal nanowires of defined length.

Ionov et al. [46] described the preparation of nanostructured stimuli-thermoresponsive polymer brushes grown via atom transfer radical polymerization initiated from the surface of microtubules made of bovine tubulin. The product obtained was characterized by AFM and fluorescence studies, and the feasibility of its response to temperature changes was demonstrated, indicating a potential application as biomolecular switch.

Juarez et al. [47] described the preparation of gold nanowires biotemplated onto the surface of lysozyme fibrils as catalysts for the chemical reduction of *p*-nitrophenol to *p*-aminophenol by NaBH_4_. The gold nanoparticle-decorated lysozyme fibrils obtained were subjected to structural analysis by TEM and X-ray diffraction (XRD) analysis, and the feasibility of their use as a catalyst was demonstrated by kinetic spectrophotometric analysis.

Leroux et al. [48] demonstrated the use of bovine insulin derived helical fibrils as a biotemplate for the helically arranged, 4–6 nm in diameter chains of silver NPs obtained by electroless deposition, which exhibit optical activity within the visible range. The product obtained was subjected to AFM, TEM and circular dichroism spectra structural analysis.

The preparation of magnetic nanowires was also described by Juarez et al. [49]. The coating of pre-assembled human serum albumin or lysozyme fibrils was readily carried out by the controlled addition of iron salts and ammonium hydroxide, leading to preferred precipitation on the fibrils acting as a biotemplate. The Fe_3_O_4_–protein hybrids thus obtained were characterized by HRTEM, XRD and Fourier transform infrared spectroscopy (FTIR) analysis, and the feasibility of their magnetization demonstrated, leading towards potential applications as MRI contrast agents.

Korkmaz et al. [50] described the calcium-dependent self-assembly of the recombinant S-layer protein SbsC derived from *Geobacillus stearothermophilus* into tubular structures serving as biotemplates for the chemical deposition of platinum. The resulting hybrid was characterized by HRTEM, SEM and fluorescence analysis, in preparation for an intended application as nanowires.

Padalkar et al. [51] described the use of a fibrilar protein (α-synuclein) template for the growth of silica and titania nanowires for biosensing applications. SiO_2_ and TiO_2_ nanowires were grown on the biotemplate by monomer polymerization. The structures obtained were characterized by TEM and field-emission scanning electron microscopy (FESEM), and the feasibility of their use for biosensors based on the enzymatic activity of acetylcholine esterase was demonstrated and characterized.

Insulin fibers were coated with palladium by electroless deposition. The nanostructure and electrical properties of the palladium hybrids thus obtained were characterized by AFM, TEM, X-ray photoelectron spectroscopy (XPS) and scanning capacitance microscopy (SCM) analysis [52]. The use of insulin fibrils as biotemplates for the deposition of platinum and their application as a catalyst for the reduction of *p*-nitrophenol by NaBH_4_ to 4-aminophenol was described by Batzli and Love [53]; see also Juarez et al. [47].

A recent contribution by Clark et al. [54] offers a promising “ultrastable biomolecular construction kit for the assembly of filamentous proteins into geometrically defined templates of controllable size and symmetry”. The kit is based on the filamentous protein γ-prefoldin derived from the hyperthermophile *Methanocaldococcus jannaschii*, and its use as the major component of a biotemplate has been constructed in combination with other protein-made connecting units (see also Slocik et al. [45]).

## 6. Viral Envelopes as Biotemplates

The highly-ordered 3D protein envelopes of viruses, especially of plant viruses and bacteriophages, offer a ‘natural’ biotemplating tool, which is available in several geometrical shapes and sizes (for related reviews, see [55,56,57,58]).

Tsukamoto et al. [59] described the synthesis of CoPt and FePt_3_ nanowires inside the central channel of tobacco mosaic virus (TMV), used as a biotemplate for electroless deposition mediated by reduction with NaBH_4_ of cobalt and platinum ions within the channel. Nanowires ranging from 50 to 100 nm were identified by HRTEM analysis, as well as by EDXS and magnetometry measurements.

Neltner et al. [60] described the catalytic production of hydrogen gas from ethanol using Rh–Ni–CeO_2_ nanocrystals biotemplated onto the surface of M13 bacteriophages, significantly improving parameters such as operational and storage stability.

Lim et al. [61] described the formation of thin palladium nanowires, formed as a coating without the addition of an external reducer, on the surface of TMV. The wires obtained, tens of nanometers in thickness and hundreds of nanometers in length, were characterized by X-ray Raman scattering (XRS).

The production of 3 nm aligned magnetic NPs within the central channel of mutated tobamovirus was described by Kobayashi et al. [62]. Energy-dispersive X-ray spectroscopy and superconducting quantum interference analysis indicated that the nanoparticles obtained consisted of a Co–Pt alloy.

Nuraje et al. [63] demonstrated the photocatalytic and photovoltaic activity of a chemically modified envelope of M13 bacteriophage-templated crystalline perovskite nanomaterials (characterized by TEM, HRTEM and XRD), with potential applications in solar energy conversion.

The controlled preparation of a 3D super lattice of metallic nanocrystals was demonstrated by Alloyeau et al. [64], combining self-assembled tomato bushy stunt virus (TBSV) and streptavidin 2D crystals as interwoven biotemplates for the controlled 3D growth of a silver nanoparticle array (characterized by TEM).

Zhou et al. [65] described the preparation of copper nanorods and nanowires, 24–26 nm in diameter (characterized by TEM, SEM and XPS), by the electroless deposition of copper on palladium ion pre-activated TMV and M13 bacteriophage surfaces.

Flexible electroactive nanomaterials were prepared by Vera-Robles et al. [66] by biotemplating, using chemically modified pVIII protein of M13 viral envelopes, affecting nucleation and the enlargement of gold or platinum NPs. Transmission electron microscopy, SEM and electrochemical analysis demonstrated the feasibility of using the structures obtained as electrodes.

Piezoelectric nanowires were biotemplated on a genetically engineered M13 bacteriophage coating protein surface, displaying triglutamate residues for the periodical positioning of Pb^2+^ cations [67]. A lattice with an accurate spacing of 0.4 nm was readily prepared, its structure characterized by HRTEM and SEM, and the feasibility of its functionality as a lead zirconate titanate (PZT) device demonstrated. Gold dendrites were co-deposited with M13 virus as a biosensor platform for nitrite ions by Seo et al. [68] and tunable core-shell barley stripe mosaic virus (BMMV)–palladium nanowires were readily obtained, as described by Adigum et al. [69].

As the structural complexity degree of biotemplated arrays grows, it should be mentioned that new characterization tools are also gradually becoming available for the biotemplating arena, as demonstrated by the recent report by Carreno-Fuentes et al. [70] on the combination of molecular docking and advanced electron microscopy, providing imaging through ultrahigh-resolution FESEM and spherical aberration-corrected scanning transmission electron microscopy (STEM) at a low voltage.

## 7. Protein Crystal-Mediated Biotemplating

**Figure 4:**
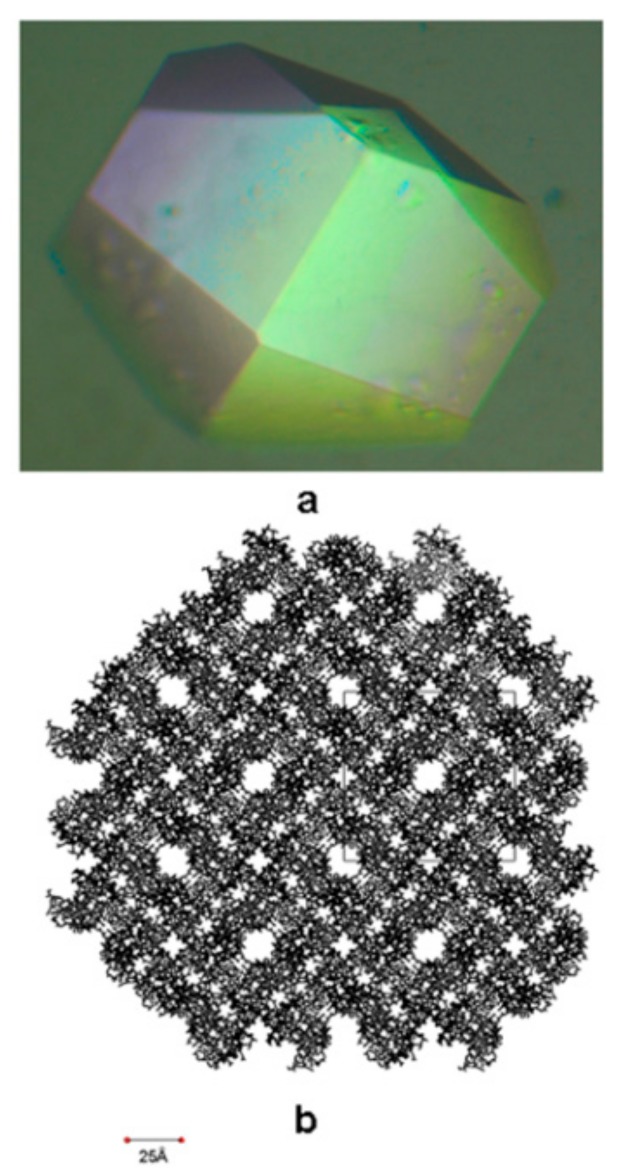
Formation of voids array in a protein crystal. (**a**) Tetragonal lysozyme crystal; (**b**) Cross-section presenting the pore-array within the lysozyme crystal. The unit cell is shown as a solid line. Reprinted with permission from [71], Copyright 2009, Begell House Inc.

Protein crystals, routinely prepared for the elucidation of protein 3D structures by X-ray crystallography, present a highly ordered 3D array of protein molecules. Along with the formation of this array, a complementary 3D array of nanosize voids is also formed; with patterns, geometry and dimensions depending on the protein ‘building block’ and its mode of ‘packaging’ within the crystal (Figure 4).

The composites obtained by ‘filling’ these voids of protein crystals as biotemplates have potential in providing nanostructured, 3D, highly accurate arrays of hybrid materials for applications in fields such as electronic devices (for a review of potential applications, see [72]) and spectroscopy (for related reviews, see [73,74,75,76]).

The exploitation of the potential inherent to 3D protein crystal biotemplating calls for the availability of complex infrastructures, including the means, know-how, and the control of protein crystallization and protein crystal stabilization to prevent crystal dissolution; as well as structural analysis by XRD and related advanced spectrophotometric methods in order to monitor the preservation of the parent 3D crystalline structure throughout the templating procedure, and the prevention of distortion resulting from the impact on the template array by the templated material.

The feasibility of using such an infrastructure for the monitoring of the stability of a crosslinked lysozyme crystal throughout biotemplating of a crosslinked hydrogel inside its internal voids array was successfully demonstrated in our laboratory [77]. Following the stabilization of the crystals by glutaradehyde, crosslinking the crystals was equilibrated with a monomer/crosslinker mixture (acrylamide/ethyleneglycol-dimethacrylate solution), with subsequent in situ polymerization. The monitoring of possible protein crystal structural changes taking place throughout the process of the biotemplating of the formed hydrogel was enabled by the combination of step-by-step comparative analysis of the data obtained from X-ray crystallography and the fluorescence decay analysis of ultrafast laser activated soluble dye indicator, confirming the preservation of the 3D structure of the parent protein crystal biotemplate throughout this process.

Stabilization of protein crystals by chemical crosslinking is a pre-requisite to controlled 3D biotemplating, as the stability of the crystalline protein template should be preserved throughout the process. This stabilization is mostly affected by glutaraldehyde crosslinking (for a review on glutaraldehyde crosslinked protein crystals and their applications, see [78]). In view of the efficacy described above in the stabilization and preservation of the lysozyme crystal template throughout the biotemplating process, we used our structural analysis infrastructure, especially the X-ray crystallography capabilities, to elucidate the mechanism of protein crystal chemical crosslinking by glutaraldehyde, identifying a leading role for polymeric forms of glutaraldehyde in the process (for details, see [79]). The preservation provided by glutaraldehyde crosslinking, enabled the 3D biotemplating of silver grains grown within the voids array of concanavalin A crystal pre-stabilized by glutaraldehyde crosslinking as was demonstrated by Cohen-Hadar et al. [71].

The elucidation of the glutaraldehyde crosslinking mechanism and an understanding of its progress throughout the protein crystal structure paved the way for the potential inherent to site-preferred partial crosslinking, enabling the isolation of the homogeneous population of protein products (e.g., protein dimmers) “liberated” from partially crosslinked crystals by re-dissolution, as demonstrated in our laboratory [80].

A large spectrum of 3D void arrays, exhibiting different shapes and sizes, is available for exploitation from the Protein Data Bank (PDB). Their use is, however, often limited by the commercial availability of an envisaged protein or tedious crystallization procedures and their structural analysis. An alternative route for fishing data out from the PDB might be the use of a “parent” protein building block for the preparation of a series of protein crystals, each exhibiting a different 3D void array. This approach may be materialized by affecting different crystallization conditions: (i) modification of salt and alcohol concentrations, (ii) “directing” the crosslinking of binding proteins by bi-ligand as demonstrated by crosslinking of concanavalin A, yielding ‘diamond-like’ crystals by crosslinking with monosaccharide bi-ligands [81]; or (iii) parent protein surface modifications of amino acids involved in protein–protein interactions leading to the ‘parent’ crystal, selected from X-ray crystallography data derived from the “parent crystal”.

**Figure 5:**
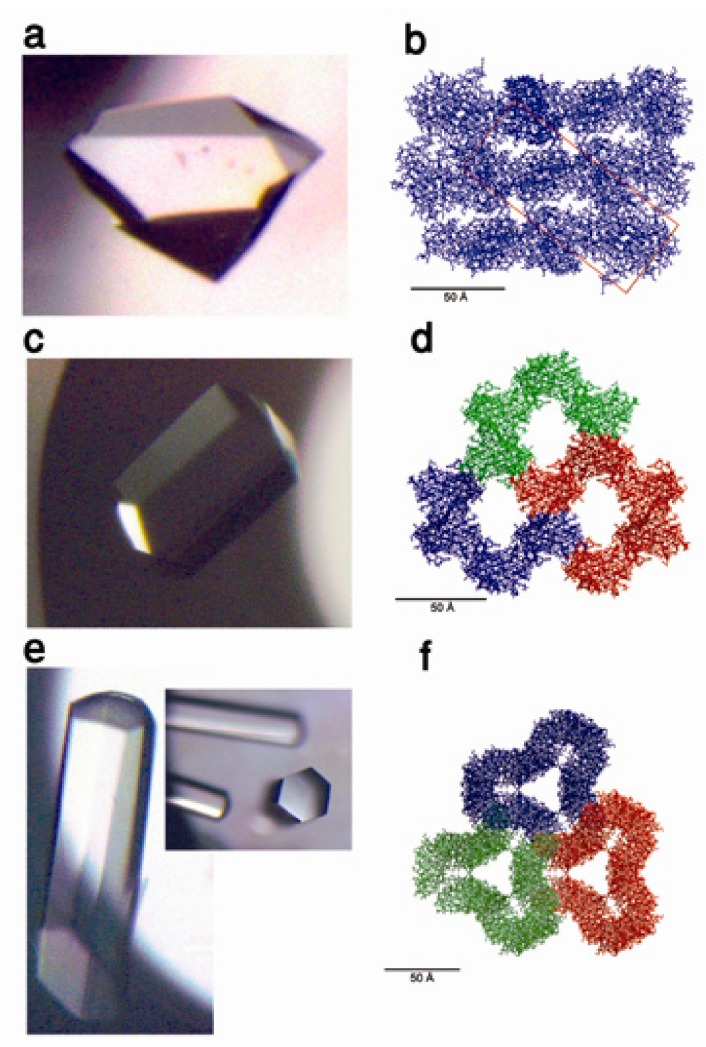
(**a**) Optical micrograph of a parent cohesin 1 (Coh1) crystal and (**b**) ribbon representation of its crystal packing (cross-section in *bc* plane). (**c**) Optical micrograph of a K42W mutant protein crystal and (**d**) ribbon representation of its crystal packing (cross-section in *bc* plane). (**e**) Optical micrograph of a K117W-K119W double mutant protein crystal and (**f**) ribbon representation of its crystal packing (cross-section in *bc* plane). Reprinted with permission from [83], Copyright 2010, AIP Publishing LLC.

To illustrate the feasibility of the latter approach, Wine and coworkers [82,83] used protein engineering tools to modify the packing of a selected protein (cohesin from *Acetivibrio cellulolyticus*) as illustrated in Figure 5.

**Figure 6:**
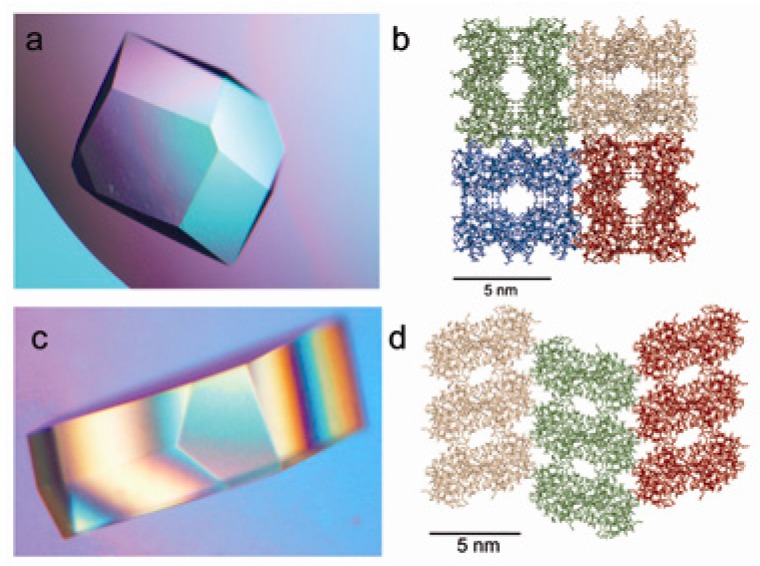
(**a**) Optical micrograph of native lysozyme tetragonal crystal and (**b**) ribbon presentation of its crystal packing (cross-section in ab plane). (**c**) Optical micrograph of maleic anhydride modified lysozyme crystal and (**d**) ribbon presentation of its crystal packing (cross-section in *ab* plane). Reprinted with permission from [83], Copyright 2010, AIP Publishing LLC.

An alternative route to affect parent protein–protein molecular interactions affecting crystal “packing” was demonstrated by Cohen-Hadar et al. [84], using the chemical modifications of the lysine amino groups of the parent protein, as shown in Figure 6 for lysozyme crystals.

Takeda et al. [85] suggested and demonstrated the feasibility of biotemplating pre-prepared gold NPs by a co-crystallization from lysozyme solution, admixed with a gold NPs suspension (the product was characterized by spectroscopy, light and electron microscopy).

He and colleagues [86,87] demonstrated the preparation and use of lysozyme crosslinked crystal-embedded silver NPs as catalysts for the chemical reduction of 4-nitrophenol to 4-aminophenol by NaBH_4_, indicating the superiority of smaller crystals for this application.

## 8. Protein-Mediated Biotemplating by Cell Fragments

The feasibility of using bacterial flagella, either extracted from bacterial cells or re-assembled from purified flagellin, was demonstrated by several groups.

Mao and colleagues [88] demonstrated the controlled synthesis of hollow double layered core-shell titania–silica nanotubes using flagella detached from *Salmonella typhimurium* (confirmed by HRTEM and EDXS structural analysis). Oxide formation on the surface of a series of biological templates, including flagella mechanically detached from *S. typhimurium* was also demonstrated by this laboratory [89], using chemical surface modification enabled the subsequent nucleation and growth of biotemplated silica (confirmed by SEM, TEM and nuclear magnetic resonance (NMR) structural analysis). The potential removal of the biotemplate from the final hybrid thus obtained offered potential applications in nanoelectronics.

Gopinathan et al. [90] demonstrated the feasibility of biotemplating silver NPs by electroless deposition on the surface of *S. typhimurium* flagella reconstructed from isolated and purified flagellin. The hybrid obtained was characterized by HRTEM, X-ray and Raman spectroscopy, followed by electrochemical I-V measurements, indicating that electrical conductivity of the hybrid prepared from reconstructed flagella was superior to the conductivity exhibited by directly extracted flagella.

## 9. Conclusions and Future Perspectives

Protein-mediated biotemplating has attracted much attention throughout the past decade, culminating in data proving the feasibility of a wide spectrum of biotemplating systems demonstrated in one, two and three dimensions. All these data are supported by advanced, state-of the-art characterization technologies.

The preliminary feasibility demonstrations of several applications made available by protein-mediated biotemplating was also demonstrated with an emphasis on nanoelectronics and biosensing. It appears, however, that most protein-mediated biotemplating systems described so far did not culminate in feasibility demonstrations for their potential applications. Therefore, it appears that protein-mediated biotemplating-related research and development (R+D) efforts should address the challenges of the identification and demonstration of new applications, and focus on further developing the already described initiations.

Future advancement of such a technology should also aim to solve important practical aspects as bringing results from bench to practice, including the provision of the means of contact or connection of the hybrids thus obtained to working/detecting systems, as well as data on storage and operational stability. The process of coping with future R+D challenges may benefit from further exploitation of the potential inherent to the incorporation of additional protein engineering and chemical modification-based methods for the manipulation and performance improvement of protein templates.

## References

[1] Lagziel-Simis S., Cohen-Hadar N., Moscovich-Dagan H., Wine Y., Freeman A. (2006). Protein-mediated nanoscale biotemplating. Curr. Opin. Biotechol..

[2] Ma N., Sargent E.H., Kelley S.O. (2008). Biotemplated nanostructures: Directed assembly of electronic and optical materials using nanoscale complementarity. J. Mater. Chem..

[3] Behrens S.S. (2008). Synthesis of inorganic nanomaterials mediated by protein assemblies. J. Mater. Chem..

[4] Galloway J.M., Stainland S.S. (2012). Protein and peptide biotemplated metal and metal oxide nanoparticles and their patterning onto surfaces. J. Mater. Chem..

[5] Yang W., Guo W., Chang J., Zhang B. (2017). Protein/peptide-terminated biomimetic synthesis of inorganic nanoparticles for biomedical applications. J. Mater. Chem B.

[6] Zan G., Wu Q. (2016). Biomimetic and bioinspired synthesis of nanomaterials/nanostructures. Adv. Mater..

[7] Dagan-Moscovich H., Cohen-Hadar N., Ophir C., Rishpon J., Shacham-Diamand Y., Freeman A. (2007). Nanowiring of the catalytic site of novel molecular enzyme–metal hybrids to electrodes. J. Phys. Chem. C.

[8] Vernick S., Dagan-Moscovich H., Porat-Ophir C., Rishpon J., Freeman A., Shacham-Diamand Y. (2009). Directed metallization of single-enzyme molecules with preserved enzymatic activity. IEEE Trans. Nanotech..

[9] Mor G., Vernick S., Dagan-Moscovich H., Dror Y., Freeman A. (2011). Novel biologically active silver-avidin hybrids. J. Phys. Chem. C.

[10] Freeman A., Dror Y., Ophir-Porat C., Hadar N., Shacham-Diamand Y. (2015). Silver coated biologically active protein hybrids: Antimicrobial applications. Appl. Mechan. Mater..

[11] Veelders M., Essen L.O. (2012). Complex gadolinium-oxo clusters formed along concave protein surfaces. ChemBioChem.

[12] Kong Y., Chen J., Gao F., Li W., Xu X., Pandoli O., Yang H., Ji J., Cui D. (2010). A multifunctional ribonuclease-A-conjugated CdTe quantum dot cluster nanosysytem for synchronous cancer imaging and therapy. Small.

[13] Chen J., Kong Y., Ruan J., Wang K., Gao F., Cui D. (2012). Protein-induced structural evolution of silver sulfide at the nanoscale: From hollow particles to solid spheres. Nanoscale.

[14] Wang Z., Chen L., Huang Y., Jia N. (2017). Albumin-mediated platinum nanocrystals for in vivo enhanced computed tomography imaging. J. Mater. Chem. B.

[15] Wang J., Yang S., Li C., Miao Y., Zhu L., Mao C. (2017). Nucleation and assembly of silica into protein-based nanocomposites as effective anticancer drug carriers using self-assembled silk protein nanostructures as biotemplates. ACS Appl. Mater. Interfaces.

[16] He M., Zhang Y., Muneyama J.C., Wu T., Yang Z., Chen H., Qu W., Xiao J. (2017). Tuning the hierarchical nanostructure of hematite mesocrystals *via* collagen-templated biominaralization. J. Mater. Chem. B.

[17] Zhang L., Knez M. (2012). Spherical nanoscale templates for biomedical applications: A review on ferritin. J. Nanosci. Lett..

[18] Jutz G., Rijn P., Miranda B.S., Boker A. (2015). Ferritin: A versatile building block for bionanotechnology. Chem. Rev..

[19] Zang J., Chen H., Zhao G., Wang F., Ren F. (2017). Ferritin cage for encapsulation and delivery of bioactive nutrients: From structure, property to applications. Crit. Rev. Food Sci. Nutr..

[20] Chen H., Zhang S., Xu C., Zhao G. (2016). Engineering protein interfaces yields ferritin disassembly and reassembly under benign experimental conditions. Chem. Commun..

[21] Eloi J.C., Ward Jones S.E., Poor V., Okuda M., Gwyther J., Schwarzacher W. (2012). Electrochemically triggered adsorption of biotemplated nanoparticles on self-assembled organometallic diblock copolymer thin films. Adv. Funct. Mater..

[22] Sano K.I., Yoshii S., Yamashita I., Shiba K. (2007). In aqua structuralization of a three-dimensional configuration using biomolecules. Nano Let..

[23] Iwahori K., Enomoto T., Furusho H., Miura A., Nishio K., Mishima Y., Yamashita I. (2007). Cadmium sulfide nanoparticle synthesis in Dps protein from *Listeria innocua*. Chem. Mater..

[24] Okuda M., Suzumoto Y., Iwahori K., Kang S., Uchida M., Douglas T., Yamashita I. (2010). Biotemplated CdSe nanoparticle synthesis in a cage shaped protein *Listeria*-Dps and their two dimensional ordered array self-assembly. Chem. Commun..

[25] Huggins K.N.L., Schoen A.P., Arunagirinathan M.A., Heishorn S.C. (2014). Multi-site funcionalization of protein scaffolds for bimetallic nanoparticle templating. Adv. Funct. Mater..

[26] Voet A.R.D., Noguchi H., Addy C., Zhang K.Y.J., Tame J.R.H. (2015). Biomineralization of a cadmium chloride nanocrystal by a designed symmetrical protein. Angew. Chem. Int. Ed..

[27] Behrens S., Heyman A., Maul R., Essig S., Steigenwald S., Quintilla A., Wenzel W., Burck J., Degany O., Shoseyov O. (2009). Constrained synthesis and organization of catalytically active metal nanoparticles by self-assembled protein templates. Adv. Mater..

[28] Zhou Z., Bedwell G.J., Li R., Prevelige P.E., Gupta A. (2014). Formation mechanism of chalcogenide nanocrystals confined inside genetically engineered virus-like particles. Sci. Rep..

[29] Maity B., Abe S., Ueno T. (2017). Observation of gold sub-nanocluster nucleation within a crystalline protein cage. Nat. Comm..

[30] Zhou Z., Bedwell J., Li R., Pachoudhury S., Prevelige P.E., Gupta A. (2017). Pathways for gold nucleation over protein cages. Langmuir.

[31] Slytr U.B., Messner P., Pum D., Sara M. (1999). Crystalline bacterial cell surface layers (S layers): From supramolecular cell structure to biomimetics and nanotechnology. Angew. Chem. Int. Ed..

[32] Mark S.S., Bergkvist M., Bhatnager P., Welch C., Goodyear L.A., Yang X., Angert E.R., Batt C.A. (2007). Thin film processing using S-layer proteins: Biotemplating assembly of colloidal gold etch mask for fabrication of silicon nanopillar arrays. Colloid. Surf. B Biointerface.

[33] Sierra-Sastre Y., Choi S., Picraux S.T., Batt C.A. (2008). Vertical growth of Ge nanowires from biotemplated Au nanoparticle catalysts. J. Am. Chem. Soc..

[34] Queitsch U., Hamann C., Schaffel F., Rellinghaus B., Schultz L., Bluher A., Mertig M. (2009). Toward dense biotemplated magnetic nanoparticle array: Probing the particle–template interaction. J. Phys. Chem. C.

[35] Varga M., Roedel G., Pompe W. Engineering of self-assembling proteins for biosensing applications. Proceedings of the 11th IEEE International Conference on Nanotechnology.

[36] Valero E., Martin M., Galvez N., Sanchez P., Raff J., Merroun M.L., Dominguez-Vera J.M. (2015). Nanopatterning of magnetic CrNi Prussian blue nanoparticles using a bacterial S-layer as biotemplate. Inorg. Chem..

[37] Shindel M.M., Mohraz A., Mumm D.R., Wang S.W. (2009). Modulating colloidal adsorption on a two- dimensional protein crystal. Langmuir.

[38] Shindel M.M., Mumm D.R., Wang S.W. (2010). Biotemplating of metallic nanoparticle arrays through site specific electrostatic adsorption on strepravidin crystals. Langmuir.

[39] Shindel M.M., Mumm D.R., Wang S.W. (2011). Manipulating energy landscapes to tune ordering in biotemplated nanoparticle arrays. Langmuir.

[40] Gao Z.D., Liu H.F., Li C.Y., Song Y.Y. (2013). Biotemplated synthesis of Au nanoparticles-TiO_2_ nanotube junctions for enhanced direct electrochemistry of heme proteins. Chem. Commun..

[41] Galloway M.J., Bramble J.P., Rawlings A.E., Burnell G., Evans S.D., Staniland S.S. (2012). Biotemplated magnetic nanoparticle arrays. Small.

[42] Bird S.M., Galloway J.M., Rawlings A.E., Bramble J.P., Staniland S.S. (2015). Taking a hard line with biotemplating: Cobalt-doped magnetite magnetic nanoparticles arrays. Nanoscale.

[43] Colby R., Hulleman J., Padalkar S., Rochet J.C., Stanciu L.A. (2008). Biotemplated synthesis of metallic nanoparticle chains on a α-synuclein fiber scaffold. J. Nanosci. Nanotech..

[44] Malisauskas M., Meskys R., Morozova-Roche L.A. (2008). Ultrathin silver nanowires produced by amyloid biotemplating. Biotechnol. Prog..

[45] Slocik J.M., Kim S.N., Whitehead T.A., Clark D.S., Naik R.R. (2009). Biotemplated metal nanowires using hyperthermophilic protein filaments. Small.

[46] Ionov L., Bocharova V., Diez S. (2009). Biotemplated synthesis of stimuli-responsive nanopatterned polymer brushes on microtubules. Soft Matter.

[47] Juarez J., Cambon A., Goy-Lopez S., Topete A., Taboada P., Mosquera V. (2010). Obtention of metallic nanowires by protein biotemplating and their catalytic application. J. Phys. Chem. Lett..

[48] Leroux F., Gysemans M., Bals S., Batenburg K.J., Snauwaert J., Verbiest T., Hasendonck C.V., Tendeloo G.V. (2010). Three-dimensional characterization of helical silver nanochains mediated by protein assemblies. Adv. Mater..

[49] Juarez J., Cambon A., Topete A., Taboda P., Mosquera V. (2011). One-dimensional magnetic nanowires obtained by protein fibril biotemplating. Chem. Eur. J..

[50] Korkmaz N., Ostermann K., Rodel G. (2011). Calcium dependent formation of tubular assemblies by recombinant S-layer protein in vivo and in vitro. Nanotechnology.

[51] Padalkar S., Schroeder K., Won Y.H., Jang H.S., Stanciu L. (2012). Biotemplated silica and titania nanowires: Synthesis, characterization and potential applications. J. Nanosci. Nanotech..

[52] Zhou X., Li R., Dai B., Zhang Y., Xu P., Zhang Y. (2013). The fabrication and electrical characterization of protein fibril-templated one-dimensional palladium nanaostructures. Eur. Polym. J..

[53] Batzli K.M., Love B.J. (2015). Formation of platinum-coated templates of insulin nanowires used in reducing 4-nitrophenol. Mater. Sci. Eng. C.

[54] Glover D.J., Giger L., Kim S.S., Naik R.R., Clark D.S. (2016). Geometrical assembly of ultrastable protein templates for nanomaterials. Nat. Commun..

[55] Young M., Willits D., Uchida M., Douglas T. (2008). Plant viruses as biotemplates for materials and their use in nanotechnology. Ann. Rev. Phytopathol..

[56] Sanghamitra N.J., Inaba H., Kitagawa S., Ueno T. (2013). Inorganic design of protein assemblies as supramolecular platforms. J. Inorg. Polym..

[57] Love A.J., Makarov V., Yaminsky I., Kalinina N.O., Taliansky M.E. (2014). The use of tobacco mosaic virus and cowpea mosaic virus for the production of novel metal nanomaterials. Virology.

[58] Steele J.F.C., Peyret H., Saunder K., Castells-Graells R., Marsian J., Meshcheriakova Y., Lomonossoff G.P. (2017). Synthetic plant virology for nanobiotechnology and nanomedicine. WIREs Nanomed. Nanobiotechnol..

[59] Tsukamoto R., Muraoka M., Seki I., Tabata H., Yamashita I. (2007). Synthesis of CoPt and FePt_3_ nanowires using the central channel of TMV as a biotemplate. Chem. Mater..

[60] Neltner B., Peddie B., Xu A., Donelen W., Durand K., Yun D.S., Speakman S., Peterson A., Belcher A.M. (2010). Production of hydrogen using nanocrystalline protein-templated catalysts on M13 phage. ACS Nano.

[61] Lim J.S., Kim S.M., Lee S.Y., Stach E.A., Culver J.N., Harris M.T. (2010). Biotemplated aqueous-phase palladium crystallization in the absence of external reducing agents. Nano Lett..

[62] Kobayashi M., Seki M., Tabata H., Watanabe Y., Yashimata I. (2010). Fabrication of aligned magnetic nanoparticles using tobamoviruses. Nano Lett..

[63] Nuraje N., Dang X., Allen M.A., Lei Y., Belcher A.M. (2012). Biotemplated synthesis of perovskite nanomaterials for solar energy conversion. Adv. Mater..

[64] Alloyeau D., Stephanidis B., Zhao X., Larquet E., Boisset N., Ricolleau C. (2011). Biotemplated synthesis of metallic nanoclusters organized in tunable two-dimensional superlattices. J. Phys. Chem. C.

[65] Zhou J.C., Soto C.M., Chen M.S., Bruckman M.A., Moore M.H., Barry E., Ratna B.R., Pehrsson P.E., Spies B.R., Confer T.S. (2012). Biotemplating rod-like viruses for the synthesis of copper nanorods and nanowires. J. Nanobiotech..

[66] Vera-robles L.I., Nhieu G.V.T., Laberty-Robert C., Livage J., Sanchez C. (2013). Flexible electroactive nanomaterials biotemplated with versatile M13 phage platforms. Adv. Eng. Mater..

[67] Cung K., Han B.J., Nguyen T.D., Mao S., Yeh Y.W., Xu S., Naik R.R., Poirier G., Purohit P.K., McAlpine M.C. (2013). Biotemplated synthesis of PZT nanowires. Nano Lett..

[68] Seo Y., Manivannan S., Kang I., Lee S.W., Kim K. (2017). Gold dendrites co-deposited with M13 virus as a biosensor platform for nitrite ions. Biosens. Bioelectron..

[69] Adigum O., Retlaff-Roberts E.L., Novikova G., Wang L., Kim B.S., Ilavsky J., Miller J.T., Loesch-Fries L.S., Harris M.T. (2017). BSMV as a biotemplate for palladium nanomaterial synthesis. Langmuir.

[70] Carreno-Fuentes L., Bahena D., Palomares L.A., Ramirez O.T., Jose-Yacaman M., Plascencia-Villa G. (2016). Molecular docking and aberration corrected STEM of palladium nanoparticles on viral templates. Metals.

[71] Cohen-Hadar N., Wine Y., Lagziel-Simis S., Moscovich-Dagan H., Dror Y., Frolow F., Freeman A. (2009). Protein crystal-mediated biotemplating. J. Porous Med..

[72] Uraoka Y., Uenuma M., Ishikawa Y., Kumagai S., Tomita S., Watanabe H., Yamashita I., Sone J., Tsuji S. (2016). Biotemplates and their application to electronic devices. Intelligent Nanosystems for Energy, Information and Biological Techniques.

[73] Dotan N., Cohen N., Kalid O., Freeman A., Rosoff M. (2001). Supramolecular assemblies made of biological macromolecules. Nano-Surface Chemistry.

[74] Rica R.D.L., Matsui H. (2010). Applications of peptide and protein based materials in bionanotechnology. Chem. Soc. Rev..

[75] Sotiropoulou S., Sierra-Sastre Y., Mark S.S., Batt C.A. (2008). Biotemplated nanostructured materials. Chem. Mater..

[76] Howorka S. (2011). Rationally engineering natural protein assemblies in nanobiotechnology. Curr. Opin. Biotechnol..

[77] Cohen-Hadar N., Wine Y., Nachlieli E., Huppert D., Gutman M., Frolow F., Freeman A. (2006). Monitoring of the stability of crosslinked protein crystals biotemplates: A feasibility study. Biotechnol. Bioeng..

[78] Yan E.K., Cao H.L., Zhang C.Y., Lu Q.Q., Ye Y.J., He J., Huang L.J., Yin D.C. (2015). Cross-linked protein crystals by glutaraldehyde and their applications. RSC Adv..

[79] Wine Y., Cohen-Hadar N., Freeman A., Frolow F. (2007). Elucidation of the mechanism and end products of glutaraldehyde crosslinking reaction by X-ray structure analysis. Biotechnol. Bioeng..

[80] Buch M., Wine Y., Dror Y., Rosenhak S., Lebendiker M., Giordano R., Leal R.M.F., Popov A.N., Freeman A., Frolow F. (2014). Protein products obtained by site-preferred partial crosslinking in protein crystals and “liberated” by redissolution. Biotechnol. Bioeng..

[81] Dotan N., Arad D., Frolow F., Freeman A. (1999). Self-assembly of a tetrahedral lectin into predesigned diamond-like protein crystals. Angew. Chem. Int. Ed..

[82] Wine Y., Cohen-Hadar N., Lamed R., Freeman A., Frolow F. (2009). Modification of protein crystal packing by systematic mutations of surface residues: Implications on biotemplating and crystal porosity. Biotechnol. Bioeng..

[83] Wine Y., Cohen-Hadar N., Lagziel-Simis S., Dror Y., Frolow F., Freeman A., Vafai K. (2010). Adjustment of protein crystal porosity for biotemplating: Chemical and protein engineering tools. Porous Media and Its Applications in Science, Engineering and Industry.

[84] Cohen-Hadar N., Lagziel-Simis S., Wine Y., Frolow F., Freeman A. (2011). Re-structuring protein crystals porosity for biotemplating by chemical modification of lysine residues. Biotechnol. Bioeng..

[85] Takeda Y., Kondow T., Mafune F. (2011). Self-assembly of gold nanoparticles in protein crystal. Chem. Phys. Lett..

[86] Liang M., Wang L., Su R., Qi W., Wang M., Yu Y., He Z. (2013). Synthesis of silver nanoparticles within crosslinked lysozyme crystals as recyclable catalysts for 4-nitrophenol reduction. Catal. Sci. Technol..

[87] Liu M., Wang L., Huang R., Yu Y., Su R., Qi W., He Z. (2016). Superior catalytic performance of gold nanoparticles within small cross-linked lysozyme crystals. Langmuir.

[88] Li D., Mathew B., Mao C. (2012). Biotemplated synthesis of hollow double-layered core/shell titania/silica nanotubes under ambient conditions. Small.

[89] Wang F., Nimmo S.L., Cao B., Mao C. (2012). Oxide formation on biological nanostructures via a structure-directing agent: Towards an understanding of precise structural transcription. Chem. Sci..

[90] Gopinathan P., Ashok A.M., Selvakumar R. (2013). Bacterial flagella as biotemplate for the synthesis of silver nanoparticle impregnated bionanomaterial. Appl. Surf. Sci..

